# Cases Extracted from the Obstetric Note-Book of the Welbeck-Street Dispensary, by Permission of Henry Davies, M.D.

**Published:** 1834-04-01

**Authors:** 


*Cases extracted from the Obstetric Note-book of the Welbeck-street Dispensary, by permission of* Henry Davies, m.d., Physician to the British Lying-in Hospital, &c.

Case i. *A copious Discharge of a Watery Fluid during Pregnancy, not followed by premature Labour.*

February, 1825. Anne Scott, æt. twenty-seven, applied at the Dispensary, stating that she feared she should be delivered even before she could reach home, as she had been discharging a clear, watery fluid, for the last three or four days, and could feel the child exactly as if it were coming into the world.

She has had two children, and has miscarried once, but has never experienced the sensations which she has now; but in her first pregnancy she had a discharge of a semitransparent fluid for about a month before delivery.

March 28th. Mr. Taylor sat up with her all night, at her request. On examination, the cervix uteri was found elongated, and the os uteri was alternately dilating and contracting; at every dilatation the patient lost at least a pint of clear fluid. No part of the child presented, and no resisting tumour could be felt on pressing the abdomen. Two injections were administered, as well as a dose of castor-oil; and on the following night a sedative was given.

July 25th. The patient continued her usual avocations, though the fluid dribbled away, until today, when Mr. Taylor was again called to her at half-past twelve P.M. She was suffering from regular labour-pains, after which she expelled a quantity of fluid. She would not consent to an examination per vaginam.

26th. The pains still continue, followed by a gush of fluid.

28th. On examination per vaginam, the head of the child was plainly to be felt presenting at the brim of the pelvis. The pains continued, though neither so strong nor so frequent, and the fluid was continually dripping from her till

August 9th, when she was delivered of a small female child, at half-past two A.M. As the placenta was not expelled by the natural efforts, Mr. Taylor passed up his hand at five o'clock, when he found it attached to the uterus by a few fibres, which were detached with great difficulty and much suffering to the patient. She was ordered not to be moved, as there was some hemorrhage, and the uterus did not contract, and to have nothing but cold toast and water.

11th. Much better. The pains are still very severe, and are increased by pressure. Pulse soft, and rather quick; the bowels have not been open since delivery. She was ordered to be fomented, and to take an aperient.

12th. Can bear pressure on the abdomen, but complains of a tightness about the head. The milk begins to flow. Bowels regular; pulse natural; tongue clean, and moist.

She continued to improve, and before the end of the month was able to do her household work.

*Observations.* The above case was attended and reported by Mr. Taylor, excepting that I examined the patient the latter end of March, after he had been up with her a few nights before. She imagined that she had become pregnant in August 1824; but she was probably mistaken, as she experienced a discharge of blood three months afterwards, most likely the catamenia, accompanied, as they sometimes are, by hemorrhage. It is probable that she became pregnant in November, and quickened in March; the latter occurrence being attended by a disposition to miscarry. The immense discharge of fluid evinces the ample provision made by nature to guard the conception from pressure. These large watery discharges not unfrequently occur during pregnancy, without being followed by the expulsion of the contents of the uterus, but their source does not seem to be clearly ascertained. By some it is thought that a portion of the decidua is separated from the surface of the uterus, from which the discharge is secreted. Others suppose that a collection of fluid may take place between the chorion and amnion, forming what has been called false waters. It has also been asked, whether it may not be a portion of amniotic fluid escaping through a slight fissure of the amnion, some distance above the os uteri, and then the farther egress be prevented by a contraction of the uterus. But, whatever theory may be formed on this point, it is a practical fact, that if moderate care be taken of the patients, they will frequently complete the full period of utero-gestation without any apparent detriment to the fœtus.

The indications for the treatment of patients under these circumstances appear to be, 1st, to remove all causes of excitement, whether general or local; 2dly, to tranquillize the system; 3dly, to keep the patient in a state of perfect rest, and in the horizontal posture, while the least discharge remains.

Mrs. Scott became pregnant again, and, having passed through the period of pregnancy without any untoward occurrence, was delivered of a healthy girl, June 9th, 1827.

Case ii. *Tedious Labour, from Adhesions in the Vagina.*

August, 1828. Hannah Ellis, æt. nineteen, of a delicate constitution, states that she was delivered of a still-born child thirteen months ago, by means of instruments, in consequence, it would seem, of defective uterine action; for she says, that, after having been in labour four days, the pains became too feeble to expel the child, and that it was then extracted with the forceps. Inability to retain the urine, and great soreness of the vagina, subsequently came on; the first continued for a few days only, the soreness for several weeks; at length she began to feel some obstruction, or what she calls "a lump," in the vagina, which made intercourse difficult, though it yielded a passage to the catamenia. About five months after her delivery, notwithstanding the above circumstances, she fell pregnant, and from that time to the present she has suffered more or less from great pain and soreness of the vagina, with leucorrhœal discharge; these symptoms have been latterly conjoined with an œdematous state of the labia.

August 9th. This morning she was attacked with slight pains in the loins, which may have been brought on by intestinal irritation, as she is labouring under diarrhœa, accompanied with griping and tenesmus. On making an examination per vaginam, the finger was obstructed about two inches from the os externum, by a firm unyielding substance, or strong membrane, completely impervious, except at the lower part, where these seemed to be a small aperture, with a thin edge, not capable of admitting the point of the finger. This obstruction was in all probability produced by cicatrization of the sides of the canal, the result of injury inflicted on its membrane by the instruments, or by the long impaction of the head in the pelvis during her last labour. There was a profuse muco-purulent discharge, and the vagina, particularly at the obstructed part, was so exquisitely sensitive, that the patient would scarcely submit to an examination.

Opiates were given to allay the intestinal irritation, and fomentations applied to the pudenda.

August 11th. The diarrhœa has ceased, but since the 9th she has been harassed with slight but frequent pains in the loins. This morning the membranes broke, and the pains have left her; she has ceased also to feel the movements of the child. The uterus has evidently subsided. On examination, the obstruction was found unaltered, and no presentation could be discovered.

August 14th. There has been no recurrence of pains since the 11th; they are now returning, but are feeble, frequent, and ineffective.

August 15th. The pains towards the evening became more forcible, and a discharge of blood was observed. The vagina is rather more relaxed. The patient is exhausted from the long continuance of the short and fruitless pains. An opiate was given, and more nourishing diet allowed.

16th. The pains are now more protracted, and attended with a slight sense of bearing down. On examination, the head was found presenting through the thick membranous adhesion of the vagina, which is exceedingly painful. At four P.M. the head was still resting on the obstruction: the pains were short, and the bearing down inconsiderable. At seven P.M. the head was suddenly protruded, though there had not been much increase of pain, and she was delivered of a still-born male child. Whether the adhesions were ruptured or dilated was not ascertained, as the expulsion of the head took place during the absence of Mr. Prout, the gentleman attending her, to whom I am indebted for the report of the case.

She recovered well, and was seen by Mr. Prout for several weeks afterwards, but refused to have any means used to effect the dilatation of the part.

Cases of this kind sometimes occur from inattention to the parts after the use of instruments, and sometimes even after a severe natural labour.

